# Factors contributing to the uptake of childhood vaccination in Galkayo District, Puntland, Somalia

**DOI:** 10.1080/16549716.2020.1803543

**Published:** 2020-08-27

**Authors:** Mohamed Farah Abdullahi, Jennifer Stewart Williams, Klas-Göran Sahlèn, Khalif Bile, John Kinsman

**Affiliations:** a Department of Research and Development, Puntland University of Science and Technology, Galkayo City, Somalia; b Department of Epidemiology and Global Health, Faculty of Medicine, Umeå University, Umeå, Sweden; c Research Centre for Generational Health and Ageing, Faculty of Health, University of Newcastle, Callaghan, Australia; d Senior National Advisor Health Systems and Policy and Board Member Somali and Swedish Researchers’ Association (SSRA), Vällingby, Sweden; e Department of Public Health Sciences, Global Health (IHCAR), Karolinska Institutet, Stockholm, Sweden

**Keywords:** immunization, immunization, immunity, infectious, child health, under five mortality, low and middle income countries, developing, Africa

## Abstract

**Background:**

As in many Sub-Saharan African countries, the health system in Somalia is not operating at the capacity needed to lift childhood vaccination coverage to ninety percent or above, as recommended by United Nations Children’s Fund. Current national estimates of coverage for the six major vaccine preventable childhood diseases range from thirty to sixty percent. Infectious disease outbreaks continue to pose significant challenges for the country’s health authorities.

**Objective:**

This important qualitative study, conducted in Galkayo District, Somalia, investigates limiting factors associated with childhood vaccination uptake from the perspective of both communities and health care workers.

**Methods:**

Qualitative information was collected through six focus group discussions with parents (n = 48) and five one-to-one interviews with health workers (n = 15) between March and May 2017, in three settings in the Galkayo District – Galkayo city, Bayra and Bacadwayn.

**Results:**

From a health system perspective, the factors are: awareness raising, hard to reach areas, negative attitudes and perceived knowledge of health workers, inadequate supplies and infrastructure, and missed vaccination opportunities. From the perspective of individuals and communities the factors are: low trust in vaccines, misinterpretation of religious beliefs, vaccine refusals, Somalia’s patriarchal system and rumours and misinformation. Parents mostly received immunization information from social mobilizers and health facilities. Fathers, who are typically family decision-makers, were poorly informed. The findings highlight the need for in-service training to enable health workers to improve communication with parents, particularly fathers, peripheral communities and local religious leaders.

**Conclusions:**

Enhancing knowledge and awareness of vaccination among parents is crucial. Fathers’ involvement is lacking. This may be boosted by highlighting fathers’ obligation to protect their children’s health through vaccination. It is also important that men engage with the wider community in decision-making and advance towards the global vaccination targets.

## Background

Vaccination, or the practice of introducing a vaccine into the body to produce immunity to an infectious disease, is a successful cost-effective public health intervention [1]. Immunization programs prevent 2.5 million child deaths world-wide each year [2,3] and are critical for reducing global child morbidity and mortality [4]. Immunization is key to attaining the United Nations Sustainable Development Goal 3.2 which aims to reduce under-five mortality in all countries to less than 25/1000 live births by 2030 [5].

Although global vaccination coverage has increased in recent decades, immunization uptake in many low-and-middle-income countries (LMICs) is still below the required herd immunity threshold, indicating a situation where a sufficient proportion of the population is immune to the respective infectious disease so as to make the sustained spread from person to person unlikely [6]. There is considerable disparity across sub-populations in their immunization coverage. Local context-specific information is therefore needed to better understand the reasons why children and infants in LMICs are not fully immunized so that interventions can be more effectively targeted [1,7].

In their systematic review of the published literature on childhood vaccination in LMICs, Rainey et al. examined 202 articles, from which 838 reasons associated with under-vaccination were identified. Of these reasons, 45% were associated with inadequacies in immunization systems, 26% were related to family characteristics, 22% were linked to negative parental attitudes and poor knowledge, and 7% to communication issues [1].

System-related factors contributing to sub-optimal childhood immunization include poorly arranged and coordinated services, supply stock-outs, inadequate tracking systems, under-trained vaccinators and lack of suitable venues [8–11]. Distance to services and travel costs are challenges for vaccination in rural and remote areas [7,12–15]. Traversing rivers and mountainous landscapes in difficult climatic conditions can inhibit access for both clients and providers [4,8,9,15].

Households with low maternal education [15,16] and poor income are less likely to have their children vaccinated [4,11,14,17]. Negative parental attitudes such as lack of trust in services and the technical competence of vaccinators [8] impact on vaccination decisions [4,7,10,16]. “Vaccine hesitancy” in both parents and communities refers to behaviour influenced by factors such as low trust in the vaccine and/or the provider, negative perceptions about the need for the vaccine and poor vaccine access [18]. Media exposure can influence parents’ knowledge of vaccination [7,11,13,14]. Social media research suggests that online information flows more freely between people who share similar sentiments and that social networks dominated by either positive or negative attitudes toward vaccination can impact on vaccination uptake [19].

Like many countries in Sub-Saharan Africa, Somalia’s health system is not operating at the capacity needed to lift childhood vaccination coverage to ninety percent or above, as recommended by United Nations Children’s Fund-UNICEF [20,21]. The main reasons for the low vaccination coverage are prolonged conflict and an underfunded health system [22–24]. Although there are regional variations, current national estimates of coverage for the six major vaccine preventable childhood diseases – tuberculosis, diphtheria, pertussis, tetanus, poliomyelitis (polio) and measles – range from thirty to sixty percent [20]. Infectious disease outbreaks continue to pose significant challenges for the country’s health authorities [25–27].

Conducting vaccination programs in Somalia is highly challenging for health authorities and workers as about twenty six percent of the population is nomadic [28,29] and large pockets of the country pose security issues due to activities by militia groups [30]. There needs to be greater understanding of the issues perceived both by communities and health care workers. The aim of this qualitative study conducted in Galkayo District, Somalia, is to investigate factors associated with childhood vaccination uptake from each of these two perspectives.

## Methods

### Study setting

Somalia is a Sub-Saharan African country located in the Horn of Africa with an estimated population of 12.3 million of whom almost 2.5. million are children aged under five [31]. According to government estimates, the population of Galkayo District exceeds 500,000 of whom 90,000 are children aged under five years [22,31,32].

**Figure 1.**
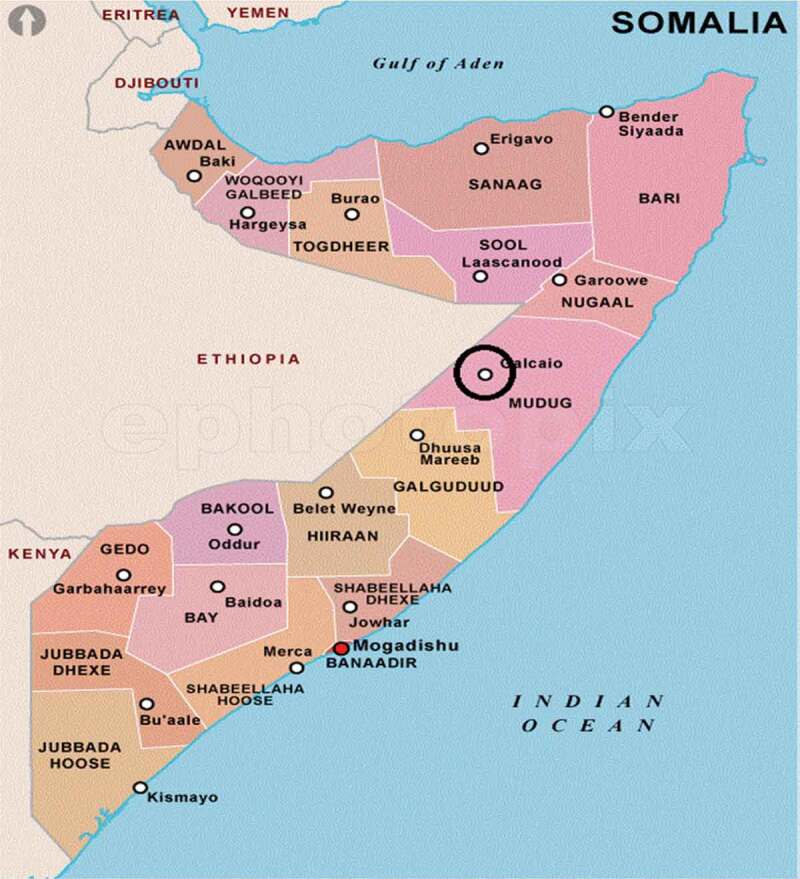
Regions of Somalia, and their capitals before the establishment of the federal government system in 2012.

This study was conducted in the northern part of the Galkayo District of Mudug Region (Figure 1) in the Puntland state. Three areas – namely Galkayo city and the villages of Bacadwayn and Bayra were targeted. The three locations were chosen because they were accessible, came under the same vaccination program and represented the demographic and geographic diversity of the District.

The Puntland Galkayo District has three public hospitals, three private hospitals and twelve health centres. These facilities are supported by the Puntland Ministry of Health and its partners such as the World Health Organization and UNICEF under the Joint Health and Nutrition Program implemented by the Save the Children Fund [33].

### Galkayo city

Galkayo city of Puntland has an estimated population of about 330,000 of whom 65,000 are under five years of age [34]. There are four hospitals (two public and two private) and six health centres providing routine vaccinations.

### Bacadwayn village

Bacadwayn is a village situated 49 kilometers north of Galkayo city with an estimated total population of 24,000 and an under-five population of 4800 as per the Puntland Ministry of Health Operational Plan. It has one public hospital and two health centres.

### Bayra village

Bayra is a village situated in the north-west of Galkayo District 27 kilometers from Galkayo city. The estimated village population is 4,500 including an under-five age population of 700 (Puntland Ministry of Health, undocumented). Bayra has one health centre that offers primary health care and routine vaccinations.

### Galkayo Puntland District vaccination services

According to the Puntland Ministry of Health (https://www.mohpuntland.com/) Galkayo District has one of the better organized health services in Somalia. The average childhood immunization coverage is estimated at sixty one percent [20,35].

Three strategies are used to reach communities. The first is a facility-based approach which serves communities close to health centres. Each centre employs five trained health workers – midwife, midwife assistant, qualified nurse, auxiliary nurse and a vaccinator. The second strategy encompasses planned outreach vaccination sessions for remote rural communities. These sessions are conducted regularly by each health centre. The third strategy involves immunization activities for polio and measles vaccination campaigns. This strategy, which aims to maintain the highest possible rates of vaccination coverage, supplements the routine Expanded Program on Immunization. In total, 248 health teams participate in mass vaccination campaigns – 180 in Galkayo, twelve in Bacadwayn, six in Bayra and 50 in other parts of the District.

## Study design

Qualitative information was collected through focus group discussions (FGDs) with parents and one-to-one in-depth interviews IDIs) with health workers. The first author (MFA), a local Somali resident, advised that IDIs would be appropriate for health workers and that parents would feel more comfortable participating in FGDs [36,37].

The FGDs were attended by MFA and a trained facilitator. Neither were personally known to participants. Interviews were conducted solely by MFA. The content of the FGD guide was analogous to the interview questions. Probing questions were used in both FGDs and IDIs. Icebreaker questions were used in the focus groups as prompts. See the Supplementary File for questions used in the FGDs and IDIs. The first author advised that, for cultural reasons, it was important to hold separate FGDs with mothers as some may have felt more comfortable speaking freely in women only groups.

District Health Officers advised that the recruitment of health staff working in a range of professional capacities would enable us to gather a broad range views. We took their advice and interviewed one representative from each of the following five professional categories: medical officer, nurse, trained birth attendant, vaccinator and midwife.

Qualitative information was collected from 48 FGDs and 15 IDIs across three settings between March and May 2017. See Table 1.

**Table 1.** Focus group discussions with parents.

Participants	Galkayo	Bacadwayn	Bayra	Total
Mothers only	8	8	8	**24**
Both parents	8	8	8	**24**
**Total participants**	**16**	**16**	**16**	**48**

## Study sampling

Both convenience and purposive sampling methods were used to recruit participants using local knowledge and advice. District Health Officers assisted with the recruitment of health workers. Parents from different geographic and demographic areas across the District were purposively recruited to allow a diversity of views and opinions. Invitations were issued face-to-face. There were no refusals or withdrawals.

## Data collection

Interview questions and focus group guides were prepared in English, translated into Somali, back-translated into English and checked. (See Supplementary File). Questions were pre-tested in two pilot FGDs and five one-to-one interviews during February 2017. All discussions were in the Somali language. The FGDs ranged from 44–80 minutes in length (average 60 minutes) and the interviews lasted from 9 to 31 minutes, with an average duration of 21 minutes.

All interviews and FGDs were audio taped. The Galkayo FGDs were held in Mudug Regional Health Office hall, the Bacadwayn FDGs were held in Bacadwayn Hospital hall, and the Bayra FGDs were held in the village health centre. The interviews were conducted in appropriate health centre settings suggested by the interviewees. There were no repeat interviews.

## Data analysis

This study takes a phenomenological approach. Audio recordings were transcribed (in Somali). A coding manual was developed with a set of a priori codes based on the literature review and local knowledge. The information collected from both the FGDs and IDIs was combined and initially coded. Additional codes were added inductively. Thematic analysis was used to interpret and analyse data. Data coding and analysis was undertaken by the first author with the assistance of the third and last authors (KS and JK respectively).

The responses of the parents and health workers were coded using Microsoft Word font colours, with specific colours corresponding to different themes. When this step was completed, codes were grouped under themes and further analysed inductively. Data from parents and health workers were triangulated to capture different perspectives of the same phenomenon. Finally, a narrative English language text was written for each theme and illustrated with relevant quotes.

## Ethical approval

Ethics approval was given by Puntland University of Science and Technology Research Ethical Committee Board and the Mudug Puntland Regional Health Office in Galkayo. All participants were aware that they had the option of withdrawal from the study at any time. Written consent was required from health workers. Both written and verbal was required for the parents.

## Results

**Figure 2.**
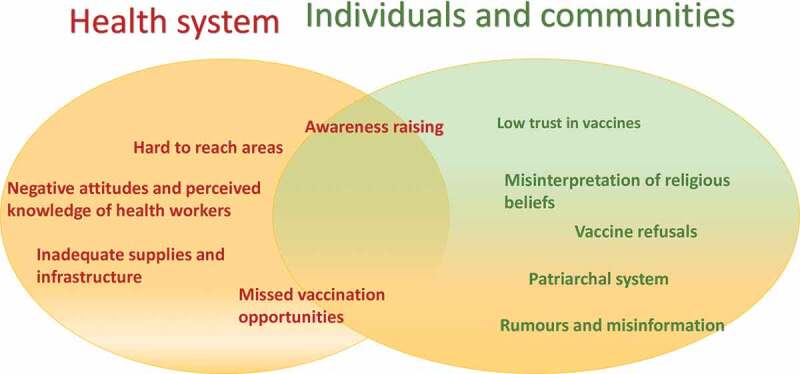
Issues related to childhood vaccination relating to the health system and/or individuals/families and communities.

The themes that emerged in the FGDs and IDIs captured both health system and community perspectives as depicted in Figure 2 and as reported below.

## Health system factors

Health system factors include: awareness raising; hard to reach areas; negative attitudes and perceived knowledge of health workers; inadequate supplies and infrastructure, and missed vaccination opportunities.

## Awareness raising

This theme refers to activities intended to raise awareness and disseminate information to parents regarding the importance of childhood immunization. Parents most often reported social mobilizers, health centres and hospitals as their main information source for immunization. See Table 2.

**Table 2.** Information sources reported by parents.

Source of information	Number of parents
Social Mobilizers	21
Health centres/hospitals	19
Radio	12
Vaccinator	9
None	5
Microphones mounted cars	4
Television	2
Community	1
Mobile messages	1

Both health workers and parents expressed the view that the current awareness raising activities were not sufficiently resourced to convince individuals in the community about the importance of vaccination. Health workers did not regard three days (as recommended by the Puntland Ministry of Health) as sufficient for these campaigns. They wanted to see more resources devoted to awareness raising, particularly for remote communities.

In one of the FGDs a mother said:

‘Lack of knowledge by parents and lack of public awareness in the community is a barrier to vaccination. If the people are told that these vaccines prevent illness and enough of this information comes from trustful health professionals, I believe that the vaccination uptake will increase.’

A clinician from Bayra commented:

‘The cause of not vaccinating those who reside here is their lack of knowledge about vaccination.’

The view was expressed that vaccine refusals were associated with negative community perceptions. A mother from Galkayo was of the view that:

‘Those who refuse vaccination complain about the lack of information about vaccination. But if they had enough information then maybe they wouldn’t refuse.’

## Hard to reach areas

Poor road infrastructure and the nomadic nature of Somali families are barriers for accessing immunization in Galkayo. The District is also prone to tribal conflicts, which can restrict the movement of health workers, specifically to and from more distant areas. One health worker from Bayra expressed the following opinion:

‘The major problem we have here are the areas where cars cannot go. Even we don’t know where the people themselves are.’

When asked how they try to overcome these challenges, one health worker explained that all that could be done was to continue to try to reach those with least access. A female vaccinator from Bayra asked:

‘What can we do then? We only try to reach those we can reach.’

A mother from Bacadwayn reflected:

‘There are two types of people, those who reside in the towns and those who reside in the countryside. The people who need the vaccination the most are those living in the countryside. But the health workers don’t have cars, they have limited time, and they will tell you themselves that they cannot always reach people. They will admit that they don’t have the time and transportation that is needed to reach everyone.’

## Negative attitudes and perceived knowledge of health workers

The attitudes and knowledge of health workers and their teams play an important role in achieving vaccination coverage. However, some parents expressed concern regarding perceived negative attitudes and knowledge by health workers. There were claims that some health workers were not well enough trained to promote immunization programs, and that some even encouraged negative perceptions of vaccines.

One vaccine-hesitant mother from Bacadwayn said that when she was a child she witnessed health workers discarding polio vaccines under trees in order to falsely record their daily targets.

‘Vaccination is something that is not good. In our childhood when vaccines were brought to the villages, we used to see vaccines poured out and dropped under trees when people refused to accept them. Remembering those days I am still suspicious and I don’t vaccinate my children.’

A nurse from Bacadwayn said:

‘There are always workers who cannot satisfy mothers. If a mother today perceives me as non-welcoming then she will not bring her child for vaccination tomorrow.’

## Inadequate supplies and infrastructure

In Bacadwayn, the hospital and two health centres rely on a third health centre for vaccine storage. The Bayra Health Centre is without cold chain storage. Vaccines stock-outs in Bacadwayn and Bayra can take days to weeks to resolve by replenishing supplies from the main cold chain situated in the city, Galkayo. Although all health centres in Galkayo city have cold chains, it was noted that there is a lack of suitable transportation for vaccines and also insufficient health workers to manage and co-ordinate the necessary tasks. Human resources are also critical. Some health workers commented that the current health workforce was unable to meet the needed immunization coverage, particularly in areas that are hard to reach.

Comments from health workers in Galkayo are reported here:

‘Currently we have measles vaccine stock-outs, and measles is just one example.’

‘Yes, we face challenges to promote our immunization program. Among the challenges we face are lack of transportation. You need transportation for example when you are getting the vaccines from the cold chain. Sometimes it happens that we go to the cold chain, obtain the vaccine, return by foot and provide the vaccines.’

## Missed vaccination opportunities

Health workers continue to find no one at home when they make house calls. One health worker made the point that if this occurs on the last day of a campaign then there is no follow-up. Challenges can also arise if the father is absent, as even if the mother is present during a house call, she will not take the decision to vaccinate her child without the father’s authorization.

A female nurse from Bayra observed:

‘A main challenge we face is taking the vaccine to the house and discovering that the mother and/or child is away. If they are at home, another problem we face is being told that the father is away and that the mother cannot make the vaccination decision. This situation occurred this week during the measles campaign.’

## Individuals and communities

The themes identified under individuals and communities include: low trust in vaccines; misinterpretation of religious beliefs; vaccine refusals; patriarchal system and rumours and misinformation. During FGDs parents were asked to reflect on their trusted sources of information. See Table 3.

**Table 3.** Trusted information sources reported by parents.

Source of information most trusted	Number of parents
Social mobilizers	16
All health workers	15
Doctor	13
Don’t trust any source	4
Community chair persons	3
Any educated person	2
Microphone	1
Radio	1
Mobile massages	1
All sources	1

## Low trust in vaccines

Parents were worried about adverse side effects post-immunization (e.g. fever and swollen legs), which they had previously observed. A mother from Bacadwayn expressed the following concerns:

‘I am so suspicious of these recurring vaccination campaigns. Even the healthiest (already vaccinated) children are being vaccinated, I have a bad perception of vaccines. I think that they contain AIDS. That is the reason I oppose vaccination’.

A male doctor from Bacadwayn contributed the following:

‘In summary, the lack of knowledge in the community and poor social awareness in general, have contributed big challenges for vaccination. These challenges can weaken our vaccine conviction.’

Some participants were worried that vaccines were used after expiry dates. Questions were raised about safety and quality control. A nurse from Bacadwayn said:

‘Sometimes they ask you who has tested the quality of the vaccines. They think that diseases are being injected into children. There are some people who think like that but they are not many.’

## Misinterpretation of religious beliefs

Some religious sects in the District overtly oppose vaccination because it is regarded as ‘sinful’. Religious and political views impact on vaccination decisions. A female nurse from Galkayo made the following comment:

‘Some of the people oppose vaccination and say that it comes from an infidel country. I believe in the Quran’

A father from Galkayo said:

‘In the name of Allah, vaccination is for curing existing diseases … I have seen sick people and animals cured from their diseases. But if I say to you that I am vaccinating you from polio that will come to you (from Allah), then maybe this is sinful. Allah knows what will come.’

## Vaccine refusals

Almost half the health workers and many parents agreed that refusals to immunize are the single largest barrier. When probed about the challenges she faced, a midwife from Bacadwayn responded:

‘*Personally I find that one of the major challenges we face is when parents refuse to accept the offer of vaccination. But some who refuse accept later when we come back*’.

A mother from Galkayo said:

‘Those who refuse vaccination complain about the lack of information about vaccination. But if they had enough information then maybe they wouldn’t refuse.’

## Patriarchal system

Participants noted that the majority of social mobilizers are female and that mothers (not fathers) are targeted during mobilization activities. However there was also a perception that targeting mothers marginalizes fathers depriving them of rights to be fully informed regarding their children’s welfare.

A mother in a FGD expressed the following opinion:

‘The first challenge to overcome in vaccinating children is the father of the family who may refuse to allow the child to be vaccinated. This is one of the reasons given for not vaccinating.’

A nurse from Bacadwayn said:

‘For the parents, the mothers may accept the vaccine, but it is the fathers who are our main challenge.’

## Rumours and misinformation

One of the vaccinators from Bacadwayn was of the view that some health workers who were not given the jobs that they had wanted, retaliated by spreading negative rumors about vaccination.

A female nurse from Bacadwayn recalled:

‘One of the challenges is that health workers are demanding jobs from the immunization programs, and if they don’t get the jobs they want, they spread rumors. They say, for example, that the jobs are only being given to those in certain tribes’.

The view was expressed by some study participants that the health system had failed to effectively engage with the community in dispelling false rumors about vaccines. Some parents believed that vaccines contain HIV or sterilization agents. An occurrence in Libya in which children were infected with HIV in a health care setting in 1998, was cited [10].

## Discussion

This qualitative phenomenological study describes health system and community related factors that contribute to the sub-optimal uptake of childhood vaccination in the Galkayo District, Somalia. The District has undergone persistent communal conflicts over recent decades and was at the epicenter of the polio outbreak in Somalia and the East African region in 2013 [26,27]. Average immunization coverage is about sixty percent [35]. Taking local circumstances into account, this study provides rich contextual information that can inform policy and decision-makers about ways of better targeting childhood vaccination in the District [18,20,38].

In spite of well-intended program efforts to increase advocacy, community engagement and health worker capacity [27] the findings reveal that the health system does not have the capacity to reach ninety percent coverage [20]. Vaccination uptake is hindered by community perceptions, beliefs and cultural norms. In addition to ensuring that vaccines are supplied to health centres in a timely manner, there is a need to inform and lift community awareness, debunk negative attitudes and behaviors and improve local integration and coordination across services [1,2,6,8,39]. Our findings resonate with other research on childhood vaccination in Sub-Saharan African countries which has identified inadequacies in program management, service delivery, logistics, vaccine supply and quality, health workers’ capacity, advocacy and communication [6].

Parents most often reported that social mobilizers were their main source of information, followed by health facilities. Participants were critical of the health system for under-resourcing awareness raising activities, which are conducted by around 248 (mostly female) social mobilizers three days prior to mass vaccination campaigns. Other research in Africa and also Asia has similarly shown that communities are not always as well-informed about vaccination programs as expected by program managers [8]. Studies in LMICs have identified poor communication between health workers and individuals, particularly among nomadic populations [9] as barriers for childhood vaccination [1,6,40,41].

Research undertaken by the European Centre for Disease Prevention and Control [18] identified mistrust in the safety and quality of vaccines as a major barrier to vaccination. Parents in our study held the perception that vaccines were being used beyond their expiry dates and that some contained dangerous harmful ingredients. Other studies have similarly identified lack of trust in health systems and vaccines as a major barrier to childhood vaccination [1]. It is essential that health authorities establish and maintain surveillance of vaccine safety and adverse event reporting [18] and communicate honestly and openly about uncertainty and risks [10,38].

Participants expressed the view that many health workers were poorly informed and therefore unable to convince parents concerning the benefits of vaccination although some are more respectful of medical doctors [42]. In Somali culture, fathers exercise the major responsibility for family decision-making. Fathers are not well informed about immunization schedules, available vaccines and vaccine preventable diseases and this leads to vaccine objection and refusal. Yet other studies in Africa identified mothers’ lack of confidence in health workers’ training and competence as a major barrier [8]. Unsupportive provider-client relationships have also been identified as reasons for defaulting from childhood immunization [11,43]. Public confidence in the immunization workforce is crucial for ensuring and sustaining vaccine coverage [1,2,38,39].

Instances were given whereby health workers and community members whose employment was negatively impacted by vaccination programs, spread damaging rumours. There was a belief by some that immunization can lead to infertility [16]. Research by UNICEF found that anti-vaccination rumours, including misinformation about HIV/AIDS, contributed to the reduced uptake of childhood vaccination in East Africa [44]. Ignorance must be contested with knowledge rather than coercion [44].

Here, as in other studies, religious beliefs were associated with negative views on vaccination [1,9]. Some Muslim sects in Galkayo District overtly oppose vaccination. UNICEF research on combatting anti-vaccination rumours in East Africa showed that when mothers seek reassurance from religious leaders about vaccination, they are not reliably given accurate information to dispel fears. Some believe that infidel enemies are promoting vaccines in order to sterilize people [18,43,44]. In 2014 religious opposition to vaccination resulted in a measles outbreak in a small village near Bacadwayn. In a study in Banadir, Somalia, respondents noted ‘punishment from God’ as a vaccination deterrent [42].

Nomadic people in Somalia are in constant search for pastures and water making it difficult for health workers to locate them during vaccination programs. Distances, terrain and lack of road infrastructure present resource challenges for vaccination coverage [8,9,41]. Other studies have highlighted hard to reach areas and nomadic lifestyles as major challenges for immunization [41]. Research in Angola identified vast unreachable areas due to poor infrastructure and also security [45,46]. Security remains an issue in Galkacyo District due to regular armed volatile clan clashes.

Health workers were discouraged by missed vaccination opportunities. This is a common problem in vaccination programs and there are many possible explanations [10]. A study in Mozambique found that spending longer than 60 minutes in travel time to health facilities was a barrier for vaccination uptake [9]. In other studies, parents reported long trekking distance, bad terrain, transportation cost, negative staff attitudes, low quality services, waiting times and vaccine stock-out as reasons [2,8,10,11]. The authors of a study in Southern Ethiopia proposed tracking systems for health facilities as a way of reducing missed vaccination opportunities [11].

Some health facilities in Bacadwayn and all in Bayra had no cold chains for vaccine storage. Studies conducted in Asian and Sub-Saharan African countries identified inadequate vaccine supply and logistics systems, insufficient human resource capacity and poorly trained and supervised managers as systemic barriers to the uptake and completion of vaccination schedules [1,2,6,8,11]. Some have suggested that the quality of health facilities may be more important than accessibility [9].

The Galkayo District is a Muslim patriarchal community in which men are the decision-makers. In Muslim culture, women are not permitted contact with non-related men. Participants questioned the rationale for having female social mobilizers targeting mothers for childhood vaccination when fathers make the final decisions. Research in Ethiopia showed that lack of paternal involvement in immunization leads to vaccine refusal [11].

Study participants attributed vaccine refusals to fathers’ negative attitudes. Parents refuse vaccinations for many reasons including low awareness, lack of understanding of the benefits, and being busy with other activities, as well as having observed minor side effects in vaccinated children [11]. In a study in Nigeria more than one third of parents refused to immunize their children because they objected, disagreed or were concerned about immunization safety [10]. Yet ideologies and prejudices run deep and the reasons why parents refuse to vaccinate their children are complex [43].

## Strengths and limitations

This study is the first of its kind to be conducted in Galkacyo District and it will contribute to better understanding of the reasons for sub-optimal vaccination uptake in the District. The choice of methods was informed by local knowledge. We were mindful of the ethical challenges in conducting the FGDs [47] and were appreciative of the local knowledge and experience of the first author, who conducted the focus groups.

However there are limitations. The study samples were not representative of the vast and diverse population. Although the results cannot be generalised to other settings, our findings were consistent with the literature on childhood vaccination in other Sub-Saharan African countries.

Discussion and interaction between participants in the mixed focus groups was not analysed, we did not collect information on participants’ educational backgrounds, and cultural gender power dynamics may have made some women hesitant to speak out in the presence of men.

## Conclusion

Information on childhood vaccination is urgently needed in Sub-Saharan African countries where coverage remains inadequate [1,2,4,14,15]. Building public trust and confidence in vaccine safety and effectiveness is crucial. It is necessary to understand, therefore, not only the factors that drive trust, but also the local perceptions of risks and benefits within different community contexts. Vaccine safety concerns must be addressed with special focus on male parents, local religious and traditional leaders. Health workers must remain accountable for any misrepresentations, whether intended or otherwise. It is critical that health workers are given appropriate training and support to address operational field challenges. The creation of trained community imbedded health workers is one way of better educating individuals and communities about the importance of childhood vaccination in Somalia.

## Supplementary Material

Supplemental Material

## Paper context

Enhancing both mothers’ and fathers’ knowledge of the importance of childhood vaccination in Somalia is crucial as is the resourcing and upskilling of health workers to address operational field challenges. There is a need to lift community awareness, debunk negative attitudes and improve the local integration and coordination of services. Vaccine safety concerns require strategic attention with focus on male parents, local religious and traditional leaders. Ignorance and rumour must be combatted with honest skillful communication.
