# Supplementary material for: Factors contributing to the uptake of childhood vaccination in Galkayo District, Puntland, Somalia
Source: Glob Health Action. 2020 Aug 27;13(1):1803543. doi: 10.1080/16549716.2020.1803543 (PMC7480419; doi:10.1080/16549716.2020.1803543)
Supplement: Supplemental Material [file ZGHA_A_1803543_SM4333.docx]

**SUPPLEMENTARY FILE**

| **In Depth Interview Questions**  Opening: - As we have talked earlier, I am here to conduct the interview to know and investigate the perceptions and feelings of health workers to suboptimal uptake of vaccination rejection in Galkacyo district. Please don’t hesitate to ask me to clarify any questions that you may not understand, but please try to give us a detail answers on these questions. Thanks  1. Please explain how the immunization program of your area is conducted?  2. What are the vaccines/antigens available in your area? Do you ever have stock-outs of vaccines? If so, please give details of how long these last, what you do to address them, etc.  3. What do your community in this area belief in Vaccination?  4. What are your feelings and perceptions about vaccination uptake in your area? What do you think are the main factors that facilitate and that reduce uptake in this community?  5. What are the main challenges that you face in conducting vaccination program in your area? Please give details and explain how you try to overcome these challenges.  6. Overall, what, if anything, do you think could be done to improve uptake?  7. Can you share with us any special events you remember in the past either positive or negative that relate to vaccination and that may reflect people’s views about vaccination in the community?  8. Where do you get your information about vaccines from? Do you trust the vaccines yourself, or do you have doubts about some of them? Details. |
| --- |
| **Focus Group Discussion Questions**  1. What are your perceptions and feelings about vaccination as general?  a. Are there specific vaccines that you are more concerned than other?  b. Have you heard of any bad experiences in relation to any particular vaccines?  2. Thinking particularly of polio vaccine, as you know the immunization coverage of our district is very low, what do you think are the main obstacles to having children vaccinated in our district?  3. What do you think makes it possible for those parents who do manage to get their children vaccinated against polio, to do so?  4. What are your sources of information regarding vaccination?  5. Which source of information do you trust or you don’t trust? Why?  6. How do you think can immunization program improved and people convinced in vaccination specially polio vaccine?  7. What are your biggest unanswered questions or concerns about immunization? |
